# Supplementary material for: Anemia and associated factors among type-2 diabetes mellitus patients attending public hospitals in Harari Region, Eastern Ethiopia
Source: PLoS One. 2019 Dec 5;14(12):e0225725. doi: 10.1371/journal.pone.0225725 (PMC6894806; doi:10.1371/journal.pone.0225725)
Supplement: S1 Questionnaire — (DOCX) [file pone.0225725.s003.docx]

**English version of the data collection tool (questionnaire and checklist)**

Anemia and associated factors among type-II diabetes mellitus patients attending in public hospitals of Harari Region, Eastern Ethiopia, 2019

Date Service area

Code Interviewer name

| **S.No.** | **Variable** | **Response** | **Skip** |
| --- | --- | --- | --- |
| **Part I: Socio-demographic characteristics** | | |  |
| 101 | Age | (in completed year) |  |
| 102 | Sex | 1. Male  2. Female |  |
| 103 | Ethnicity | 1. Oromo  2. Adere  3. Amhara  4. Tigrie  5. Others (specify) _______________ |  |
| 104 | Religion | 1. Muslim  2. Orthodox  3. Protestant  4. Others (specify) ________________ |  |
| 105 | Marital status | 1. Single  2. Married  3. Divorced  4. Widowed  5. Separated  6. Others (specify) ____________ |  |
| 106 | Educational status | - 1. Unable to read and write   2. Able to read and write   3. Primary level (1-8)   4. Secondary level (9-12)   5. College and above |  |
| 107 | Occupation | 1. Farmer 2. Housewife 3. Merchant 4. Governmental employer 5. Others (specify) ________________ |  |
| 108 | Residence | 1. Urban  2. Rural |  |

| **Part II: Lifestyle** | | |  |
| --- | --- | --- | --- |
| 201 | Have you ever smoked cigarette? | 1. Yes  2. No | If no, skip to 204 |
| 202 | If yes, are you smoking cigarette currently? | 1 Yes  2 No | If no, skip to 204 |
| 203 | How many packets do you smoke daily? | 1. ¼ packet 2. ½ packet 3. 1 packet 4. >1 packets |  |
| 204 | Have you ever drunk alcohol? | 1. Yes  2. No | If no, skip to 208 |
| 205 | If yes, are you drinking alcohol currently? | 1. Yes  2. No | If no, skip to 208 |
| 206 | How many times do you drink alcohol in a week? | 1. Every day a week  2. Once a week  3. Twice a week  4. Three times a week  5. Four times a week  6. 5-6 times a week |  |
| 207 | How many bottle/cup are you drinking in typical days? | 1. 1-2  2. 3-4  3. 5-6  4. >6 |  |
| 208 | Do you usually do some physical activity for at least 30 minutes? | 1. Yes  2. No | If no, skip to 301 |
| 209 | If yes, about how many times a week? | 1. Once a week  2. Twice a week  3. Three times a week  4. Four times a week  5. Five times a week or more |  |
| 210 | What types of exercise are you doing (more than one answer is possible)? | 1. Brisk walking (outside or inside on a tread mill)   2. Jogging/Running  3. Dancing  4. Swimming  5. others (specify) _________ |  |

| **Part III: Household food consumption** | | | | | | |
| --- | --- | --- | --- | --- | --- | --- |
|  | In the past 7 days, how often have you eaten (in days); | Never | <1/wk | 1-2/wk | Often  (3-6/wk) | Always  (Every day) |
| 301 | Any food made from grains; injera, teff, millet, sorghum, maize, rice, wheat, bread, biscuits, or any other grain product or any food made from tubers potatoes, sweet potatoes, carrots, or other foods made from roots or tubers? |  |  |  |  |  |
| 302 | Any pulses (beans, lentils, peas, nuts)? |  |  |  |  |  |
| 303 | Any vegetables? |  |  |  |  |  |
| 304 | Any fruits? |  |  |  |  |  |
| 305 | Any meat: beef, lamb, goat, fish, chicken, liver, kidney, or other organ meats? |  |  |  |  |  |
| 306 | Any eggs? |  |  |  |  |  |
| 307 | Any dairy products; milk, cheese, yogurt (not including butter)? |  |  |  |  |  |
| 308 | Any sugar or honey? |  |  |  |  |  |
| 309 | Any oil, fat, or butter? |  |  |  |  |  |

| **Part IV: Complication, comorbidity and glycemic control related variables** | | | | | | | | | |
| --- | --- | --- | --- | --- | --- | --- | --- | --- | --- |
| 401 | | How long have you had diabetes? | | Months ______________  Years _______________ | |  | |  | |
| 402 | | Are you taking medication for diabetes? | | 1. Yes  2. No | |  | | If no, skip to 404 | |
| 403 | | If yes, which drugs/ medications are you taking? (more than one answer is possible) | | 1 Metformin  2 Metformin + Glibenclamide  3 Insulin  4 Others (specify) _________ | |  | |  | |
| 404 | | Do you take any medications other than diabetes mellitus drugs? | | 1 Yes  2 No | |  | | If no, skip to 409 | |
| 405 | | If yes, for which disease? | | Specify ___________ | |  | |  | |
| 406 | | What types of drug are you taking? | | Specify ________________ | |  | |  | |
| 407 | | Any other medication? | | 1.Yes  2. No | |  | | If no, skip to 409 | |
| 408 | | What types of drug? | | Specify ________________ | |  | |  | |
| 409 | | Do you have any loss of sensation in your feet or toes including burning, tingling or numbness (neuropathy)? | | 1 Yes  2 No | |  | |  | |
| 410 | | Do you have a history of problem with eyes (retinopathy) told by health professional? | | 1 Yes  2 No | |  | |  | |
| 411 | | Do you have a history of any kidney problem (nephropathy)? | | 1 Yes  2 No | |  | |  | |
| 412 | | Do you have any history of heart problem and blocked arteries in legs (Marco-vascular complications) | | 1 Yes  2 No | |  | |  | |
| 413 | | Do you have a history of foot sores that do not heal? | | 1 Yes  2 No | |  | |  | |
| 414 | | Have you ever been told by a doctor or nurse that you have high blood pressure? | | 1 Yes  2 No | |  | |  | |
| 415 | | Have you ever been tested for HIV/AIDS? | | 1 Yes  2 No | |  | | If no, skip to 417 | |
| 416 | | What is your result? | | 1 Positive  2 Negative | |  | |  | |
| 417 | | Do you have history of any blood loss for the last 3 months? | | 1 Yes  2 No | |  | | If no, skip to 420 | |
| 418 | | If yes, did you take medication for blood loss? | | 1 yes  2 No | |  | | If no, skip to 420 | |
| 419 | | What type of medication? | | Specify ______________ | |  | |  | |
| 420 | | Have you ever been told by a doctor or nurse that you have kidney problem for the last 3 months (CKD)? | | 1 Yes  2 No | |  | |  | |
| 421 | | Do you know your most recent blood glucose result measured after no caloric intake for at least 8 hours (FBS)? | | If yes, mg/dl  If no, refer card | |  | |  | |
| 422 | | Do you know your most recent HbA1C (glycosylated hemoglobin) result? | | If yes (in %)  If no, refer card | |  | |  | |
| **Part V: Anthropometric, blood pressure, and hemoglobin data** | | | | | | | | | |
| 501 | | Weight | | (in kg) | |  | |  | |
| 502 | | Height | | (in meter) | |  | |  | |
| 503 | | Waist circumference | | (in cm) | |  | |  | |
| 504 | | Hemoglobin result | | (g/dl) | |  | |  | |
| 505 | | Blood pressure | | mm/Hg | |  | |  | |
