# Supplementary material for: Anemia and associated factors among type-2 diabetes mellitus patients attending public hospitals in Harari Region, Eastern Ethiopia
Source: PLoS One. 2019 Dec 5;14(12):e0225725. doi: 10.1371/journal.pone.0225725 (PMC6894806; doi:10.1371/journal.pone.0225725)
Supplement: S2 Questionnaire — (DOCX) [file pone.0225725.s004.docx]

**Amharic version of the data collection tool (questionnaire and checklist)**

ደም አነሰ እና ከደም አነሰ ጋር የተገናኙ በሁለተኛዉ አይነት ሰኳር በሽታኞች ሐረር ክልል በመንግሰት ሆሰፒታሎች የሚከታተሉት ላይ ጥናት ማድረግ ነው።

የተቋሙ ሰም የመጠይቁ መለያ ቁጥር _______________________________________

መጠይቁ የተካሄደበት ቀን ____________________________________ መጠይቁን የሞላው ሰው ሰም _________________________________

| **ተ.ቁጥር** | **ጥያቄ** | **ምላሽ** | **ወዴ ለላ ጥያቄ መሄድ** |
| --- | --- | --- | --- |
| **ክፍል አንድ፡ ማህበራዊ የሰነህዝብ አወቃቀር መጠይቅ** | | |  |
| 101 | ዕድሜ? | አመት |  |
| 102 | ፆታ | 1 ወንድ  2 ሴት |  |
| 103 | ብሔርዎ ምንድነው? | 1 ኦሮሞ  2 አደረ  3 አማራ  4 ትግራይ  5 ለላ ካለ ይግልፁ__________________ |  |
| 104 | ሃይማኖትዎ ምንድነው ? | 1 ሙሰሊም  2 ኦርቶዶክሰ  3 ፕሮቴሰታንት  4 ለላ ካለ ይግልፁ_____________ |  |
| 105 | የጋብቻ ሁኔታ? | 1 ያላገባ/ች  2 ያገባ/ች  3 የሞተበት/ባት  4 የተለያዩ |  |
| 106 | የትምህርት ደረጃዎ እሰከምን ድረሰ ነው? | 1 ያልተማረ/ች  2 ያልተማረ/ች ግን መጻፍ እና ማንበበ የሚችል  3 አንደኛ ደረጃ የተማረ/ች(1-8)  4 ሁለተኛ ደረጃ የተማረ/ች （9-12）  5 ኮሌጅና ከዚህያ በላይ የተማረ/ች |  |
| 107 | ሰራዎት ምንድነው? | 1 ገበሬ  2 የቤት እመቤት  3 ነጋዴ  4 የመንግሰት ሰራተኛ  5 ለላ ካለ ይግልፁ__________________ |  |
| 108 | የሚኖሩበት ቦታ? | 1. ከተማ 2. ገጠር |  |
| **ክፍል ሁለት፡ ከኑሮ ሁኔታ ጋር የተገናኙ መጠይቅ** | | |  |
| 201 | ሰጋራ አጭሶ ያውቃሉ? | 1 አዎ  2 አይ | አይ ከሆነ ወደ 204 ይሂዱ |
| 202 | መልሶዎ አዎ ከሆነ አሁነ ያጨሳሉ? | 1 አዎ  2 አይ | አይ ከሆነ ወደ 204 ይሂዱ |
| 203 | አዎ ከሆነ በቀን ምን ያክል ፓከት ያጨሳሉ? | 1 ¼ ፓከት 2 ½ ፓከት  3 1 ፓከት 4 > 1 ፓከት |  |
| 204 | የአልኮለ መጠጥ ጠጥቶዎ ያውቃሉ? | 1 አዎ  2 አይ | አይ ከሆነ ወደ 208 ይሂዱ |
| 205 | አዎ ከሆነ አሁነ የአልኮለ መጠጥ ይጠጣሉ? | 1 አዎ  2 አይ | አይ ከሆነ ወደ 208 ይሂዱ |
| 206 | አዎ ከሆነ በሳምንት ምን ያህል ጊዜ የአልኮለ መጠጥ ይጠጣሉ? | 1 በየቀኑ  2 በሳምንት አንዴ  3 በሳምንት ሁለቴ  4 በሳምንት ሶሰቴ  5 በሳምንት አራቴ  6 በሳምንት 5_6 ጊዜ |  |
| 207 | የአልኮለ መጠጥ በምጠጡበት ቀን ምን ያህል ጠርሙሰ/ብርጭቆ ይጠጣሉ? | 1 1-2  2 3-4  3 4-5  4 > 6 |  |
| 208 | ቢያንሰ ለሰላሳ ደቂቃ ያክል የሰውነት እንቅሰቃሴ አድርጎ ያውቃሉ? | 1 አዎ  2 አይ | አይ ከሆነ ወደ 301 ይሂዱ |
| 209 | አዎ ከሆነ በሳምንት ሰንት ጊዜ ያደረጋሉ? | 1 በሳምንት አንዴ  2 በሳምንት ሁለቴ  3 በሳምንት ሶሰቴ  4 በሳምንት አራቴ  5 በሳምንት አምሰት ጊዜና ከዚያ በላይ |  |
| 210 | ምን ዓይነት እንቅሰቃሴ ነው የሚያደረጉት (ከአንድ መለሰ በላይ ይቻላል)? | 1 የእግር ጉዞ ማድረገ（ደረጃ መውጣትና መውረድ）  2 ሩጫ  3 መደነሰ  4 መዋኘት  4ለላ ካለ ይግልፁ__________________ |  |

| **ክፍል ሶሰት: የቤተሰብ አመጋገብ ሁኔታ የምግብ መጠን የሚያሳይ መጠይቅ** | | | | | | |
| --- | --- | --- | --- | --- | --- | --- |
|  | ባለፈዉ ሰባት ቀን ዉሰጥ ምን ያህል ጊዜ ነው የተመገቡት? | በጭራሽ | <1/በሳምንት | 1_2/በሳምንት | 3-6/ በሳምንት | ሁልጊዘ  (በቀን) |
| 301 | ማንኛውም አይነት የሰብል ምግቦች ፤በቆሎ፣ማሽላ፣ገብስ፣ ጤፍ፣ ስንዴ፣ሩዝ፣ ከማሽላ፣ከበቆሎ፣ከስንዴ፣ከገብስ፣ከጤፍ የተሰራ ምግብ (ለምሳሌዳቦ፣እንጀራ፣ብስኩት፣ገንፎ፣ቂጣ፣ቆሎ) / ካሮት፤ ስኳር ድንች፤ ድንች እና ከነዚህ የተሰሩ? |  |  |  |  |  |
| 302 | ማንኛውም አይነት ጥራጥሬ(ባቄላ፤አኩሪ አተር፤ሽምብራ፤ምስር፤ እና ከነዚህየተሰሩ ? |  |  |  |  |  |
| 303 | ማንኛውም አይነት አትክልት? |  |  |  |  |  |
| 304 | ማንኛውም አይነት ፍራፍሬ? |  |  |  |  |  |
| 305 | ማንኛውም አይነትሥጋ፤ የበሬ፤የበግ፤የፍየል፤የዶሮ፤ አሳ፤ጉበት፤ ኩላሊት እና ልብ? |  |  |  |  |  |
| 306 | ማንኛውም አይነት እንቁላል? |  |  |  |  |  |
| 307 | ማንኛውም አይነት የወተት ተዋጽኦ ፤ ወተት፤አይብ እና እርጎ እና ሌሎች (ከቅቤ በሰተቀር)? |  |  |  |  |  |
| 308 | ማንኛውም አይነት ስኳር፤ማር? |  |  |  |  |  |
| 309 | ማንኛውም አይነት ዘይት፤ ቅቤ፤ በቅቤ የተሰራ ምግብ? |  |  |  |  |  |

| **ክፍል አራት፡ የሰኳር በሽታ ተጓዳኝ እና ከሰኳር መጠን ቁጥጥር ጋር የተገናኙ መጠይቅ** | | | | |
| --- | --- | --- | --- | --- |
| 401 | በደምዎ ውሰጥ ሰኳር መኖኑሩን ያውቁት መቼ ነበር? | ___________ ወር  __________ ዓመት |  |  |
| 402 | ለሰኳርዎ መድሃኒት የወሰዱ ነው? | 1 አዎ  2 አይ |  | መለሶ አይ ከሆነ ወደ 404 ይህዱ |
| 403 | አዎ ከሆነ ምን አይነት መድሃኒት የወሰዱ ነው (ከአንድ መለሰ በላይ ይቻላል)? | 1 ሜትፎርምን  2 ሜትፎርምን + ግልብንክላማይዲ  3 እኒሱሊን（insulin)  4 ለላ ካለ ይግልፁ__________________ |  |  |
| 404 | ከሰኳር ህመም መድሃኒት በተጨማረ ልላ መድሃኒት ይወሰዳሉ? | 1 አዎ  2 አይ |  | አይ ከሆነ ወደ 407 ይህዱ |
| 405 | አዎ ከሆነ ለየትኛው በሽታ ነው የሚወሰዱት? | የበሽታውን ሰም ይግልፁ__________________ |  |  |
| 406 | ምን ዓይነት መድሃኒት ነው የሚወሰዱት? | የመድሃኒት ሰም ይግልፁ__________________ |  |  |
| 407 | ከዚህ ለላ መድሃኒት ይወሰዳሉ? | 1 አዎ  2 አይ |  | አይ ከሆነ ወደ 409 ይህዱ |
| 408 | ምን ዓይነት መድሃኒት ነው የሚወሰዱት? | የመድሃኒት ሰም ይግልፁ__________________ |  |  |
| 409 | ሰውነዎት ላይ ወይንም እግሮዎት/እጆዎት ላይ የማቃጠል እና የመደነዘዝ ሰሜት አለ? | 1 አዎ  2 አይ |  |  |
| 410 | ከዚህ በፊት በጤና ባላሙያ/በለላ ሰው የዓይን ችግር/ህመም አለቦዎት ተበሎ ተነግሮዎት ያውቅ ነበር? | 1 አዎ  2 አይ |  |  |
| 411 | ከዚህ በፊት የኩላሊት ህመም/እንፈክሺን አለ ተበሎ ተነግሮዎት ያውቅ ነበር? | 1 አዎ  2 አይ |  |  |
| 412 | ከዚህ በፊት በጤና ባላሙያ የልብ ህመም ፤የደም ሰር ችግር እና የተገናኘ ህመም አለቦዎት ተበሎ ተነግሮዎት ያውቃል? | 1 አዎ  2 አይ |  |  |
| 413 | እግሮዎት ላይ ሳይድን የቆየ ቁሰል ነበር? | 1 አዎ  2 አይ |  |  |
| 414 | ከዚህ በፊት በጤና ባላሙያ ደም ግፊት አለቦዎት ተበሎ ተነግሮዎት/ መድሃኒት ተሰጥጦዎት ያውቃል? | 1 አዎ  2 አይ |  |  |
| 415 | ከዚህ በፊት ለኤች አይቭ ተመርምረዎ/ አለቦዎት ተበሎ ተነግሮዎት ያውቃሉ? | 1 አዎ  2 አይ |  | መለሶ አይ ከሆነ ወደ 417 ይህዱ |
| 416 | አዎ ከሆነ ውጤቱ ምን ነበር? | 1 ፖሰትቭ  2 ነገትቭ |  |  |
| 417 | ባለፈው ሶሰት ወር ውሰጥ የደም መፍሰሰ ህመም/ ችግር አጋጥሞዎት ነበር? | 1 አዎ  2 አይ |  | አይ ከሆነ ወደ 420 ይህዱ |
| 418 | አዎ ከሆነ ለደም መፍሰሰ ህመምዎ መድሃኒት የወሰዱት አለ? | 1 አዎ  2 አይ |  | አይ ከሆነ ወደ 420 ይህዱ |
| 419 | ምን ዓይነት መድሃኒት ነው የወሰዱት? | የመድሃኒት ሰም ይግልፁ__________________ |  |  |
| 420 | ከዚህ በፊት በጤና ባላሙያ ሶሰት ወር/ከሶሰት ወር በላይ የቆየ የኩላሊት ህመም አለቦዎት ተበሎ ተነግሮዎት ነበር? | 1 አዎ  2 አይ |  |  |
| 421 | በቅርብ ጊዜ ምግብ ከበሉ ከሰምንት ሰዓት በኋላ የተሰራውን የደም ሰኳርዎን መጠን ያውቃሉ (FBS)? | 1 አዎ ሚሌግራም/ደሲሊትር  2 አይ ከሆነ የበሽተኛው ካርድ ይመለከቱ |  |  |
| 422 | በቅርብ ጊዜ የተሰራውን ሀሞግሎብን ኤ1ሲ (HgA1C) ያሰታዉሉ? | 1 አዎ %  2 አይደለም ከሆነ የበሽተኛው  ካርድ ይመለከቱ |  |  |

| **ክፍል አምሰት፡ የአንትሮፖሜትር እና የደም ናሙና ውጤት** | | | | |
| --- | --- | --- | --- | --- |
| 501 | ክብደት | ___________ ኪሎ ግራም |  |  |
| 502 | ቁመት | ____________ ሰንትሜትር |  |  |
| 503 | የወገብ ዙረያ ልከት | ____________ ሰንትሜትር |  |  |
| 504 | ሄሞግሎብን/የደም ናሙና ውጤት | ____________ግራም/ደሲሊትር |  |  |
| 505 | የደም ልኬት (BP) | _____________mmHg |  |  |
