# Supplementary material for: Anemia and associated factors among type-2 diabetes mellitus patients attending public hospitals in Harari Region, Eastern Ethiopia
Source: PLoS One. 2019 Dec 5;14(12):e0225725. doi: 10.1371/journal.pone.0225725 (PMC6894806; doi:10.1371/journal.pone.0225725)
Supplement: S3 Questionnaire — (DOCX) [file pone.0225725.s005.docx]

**Afan Oromo version of the data collection tool (questionnaire and checklist)**

Hanqina dhiigaa fi rakkoolee isaan wal qabatan dhukkubsattoota sukkaaraa gosa 2ffaa qaban kanneen hoapitaalota naannoo Harar keessatti hordoffiirra jiran, 2018/2019

Guyyaa bakka tajaajila _________________________

Code maqaa gaafatamaa ____________________________

| **Lak** | **Variables** | **Deebii** | **Gara gaaffii itti aanutti darbi** |
| --- | --- | --- | --- |
| **Kutaa I: Haala hawaasumma fi demografii** | | |  |
| 101 | Umurii | (Waggaadhaan) |  |
| 102 | Saala | 1 dhiiraa 2 dhalaa |  |
| 103 | Saba | 1 Oromoo 2 Aderee  3 Amaaraa 4 Tigiree  5 kan biro--------- |  |
| 104 | Amantii | 1 Musliima 2 Orthodoksii  3 Protestantii 4 kan biroo________________ |  |
| 105 | Haala gaa'ilaa | 1 hin heerumne 2 fuudhe/heerumte  3 Addaan bahan  4 inni/isheen lubbuun hin jiru  5 gargar bahanii jiru |  |
| 106 | Sadarkaa barnootaa | 1 barreessuu fi dubbisuu hin danda'an  2 dubbisuu fi barreessuu ni danda'a  3 Sadarkaa 1ffaa(1-8)  4 Sadarkaa 2ffaa (9-12)  5 12+ (koollejjii/ universitii/ |  |
| 107 | Gosa hojii | 1 Qotee bulaa 2 Haadha manaa  3 Daldalaa/ttuu 4 Hojjetaa mootummaa  5.kan biroo_____ |  |
| 108 | Bakka jireenyaa | 1 magaalaa 2baadiyyaa |  |

| **Kutaa II: Haala amala jireenyaa** | | |  |
| --- | --- | --- | --- |
| 201 | Jimaa in qamaataa? | 1 eyyen  2 lakki | Yoo lakki jette 204tti darbi |
| 202 | Yoo eeyye jette tamboo ni xuuxxaa? | 1 eyye  2 lakki | Yoo lakki jette 204tti darbi |
| 203 | Guyyaatti pakeeta meeqa xuuxxa? | 1 ¼ pakeetii 2 ½ paakeetii  3 1 paakeetii 4>1 paakeetii |  |
| 204 | Alkoolii dhugdee in beektaa? | 1 eeyyen  2 lakki | Yoo lakki jette 208tti darbi |
| 205 | Yoo eeyyen jette amma ni dhugdaa? | 1 eyyen  2 lakki | Yoo lakki jette 208tti darbi |
| 206 | Torban tokko keessatti yeroo meeqa dhugda? | 1 torbanitti tokko  2 torbanitti lama  3 torbanitti sadii  4 torbanitti afur  5 guyyaadhaan |  |
| 207 | Yeroo dhugdutti qaruura meeqa dhugda? | 1 1-2  2 3-4  3 5-6  4 >6 |  |
| 208 | Yeroo yerootti hojii qaamaa ni hojjettaa yoo xiqqaate daqiiqaa 30f? | 1 eeyyen  2 lakki | Yoo lakki jette 301tti darbi |
| 209 | Yoo eeyye jette torbanitti yeroo meeqa hojjetta? | 1 torbanitti tokko  2 torbanitti lama  3 torbanitti sadii  4 torbanitti afur  5 torbanitti shanii fi isaa ol |  |
| 210 | Hojii/gocha/ akkami hojjettaa (deebii tokkoo ol ni danda'ama)? | 1 miilaan ykn konkolaataan deddeemuu  2 utaalcha/fiigicha  3 shaggooyyee/qaamaan sirbuu  4 bishaan daakuu  5 kan biroo |  |

| **Kutaa III: Safartuu nyaata manaa** | | | | | | |
| --- | --- | --- | --- | --- | --- | --- |
|  | Guyyaa turban darban keessatti yeroo hagam nyaata nyaattan. | Homaa | <1/torban | 1-2/torban | often  3-6/torban | Guyyaadhaan |
| 301 | Nyaata midhaanii kamillee—biddeena, xaafii, millet, mishingaa, boqqoolloo, ruuzii, qamadii, daabboo, biskuuttii, dinnicha, dinnicha sukkaaraa, karrotii? |  |  |  |  |  |
| 302 | Nyaata dheedhii (baaqela, lentils, atara)? |  |  |  |  |  |
| 303 | Kuduraa gosa kamuu? |  |  |  |  |  |
| 304 | Fuduraa gosa kamuu? |  |  |  |  |  |
| 305 | Gosa foonii: foon booyyee, hoolaa, re`ee, qurxummii, lukkuu, tiruu, kkf? |  |  |  |  |  |
| 306 | Killee gosa kamuu? |  |  |  |  |  |
| 307 | Nyaata aannani irraa argamu—aannan, baaduu, calallii baaduu (kan dhadhaa hin qabne)? |  |  |  |  |  |
| 308 | Sukkaara ykn demma? |  |  |  |  |  |
| 309 | Dhadhaa, fatii, zayitii? |  |  |  |  |  |

| **Part IV: miidhaa fi rakkoolee walxaxoo dhukkuba sukkaaraa waliin walqabatan** | | | | |
| --- | --- | --- | --- | --- |
| 401 | Dhukkubni sukkaaraa kun erga sirratti mullate hagam ta'e? | _________ji'a  ______ (waggaa) |  |  |
| 402 | Dawaa dhukkuba sukkaaraa fudhachaa jirtaa? | 1 eeyye  2 lakki |  | Yoo lakki jette 409tti darbi |
| 403 | Dawaa fudhachaa jirtu (deebii tokkoo ol ni danda'ama). | 1 Metformin  2 Metformin + gliblenclamide  3 Insulinii  4kan biroo___________ |  |  |
| 404 | Dawaa biraa kan sukkaaraa alatti kan fudhattu ni jira? | 1 eeyyen  2 lakki |  | Yoo lakki jette 409tti darbi |
| 405 | Yoo eeyye jette, dawaa maalii? | Adda baasi |  |  |
| 406 | Dawaa gosa maaliiti? | Adda baasi |  |  |
| 407 | Dawaa biraa yoo jiraate? | 1 Eyyen  2 lakki |  |  |
| 408 | Dawaa gosa maalii? | Adda baasi |  |  |
| 409 | Qaamakee irratti miirri dhagahamuu diduu muldhateeraa, miilakee, ykn qubakee sirratti kan akka qaama si gubuu, hadooduu? | 1 eyyen  2 lakki |  |  |
| 410 | Rakkoo ijaa waliin wal qabate kan mana yaalaatti sitti himame niqabdaa? | 1 eyye  2 lakki |  |  |
| 411 | Rakkoo kalee wqliin wal qabatan ni qabdaa? | 1 eyye  2 lakki |  |  |
| 412 | Rakkoo onnee waliin walqabatan cufamuu ujummoo dhiigaa miilaa ni qabdaa (Marco vascular complications)? | 1 eyyen  2 lakki |  |  |
| 413 | Kanaanduratti madaa miila keessan irraatti mul`ate siif fayyuu dide ni jiraa? | 1 eyyen  2 lakki |  |  |
| 414 | Dhiibbaa dhiigaa qabda jedhamee waanti mana yaalaatti sitti himame ykn dawaa dhiibbaa dhiigaa fudhatte ni jiraa? | 1 eeyye  2 lakki |  |  |
| 415 | HIV/AIDS qoratamtee beektaa? | 1 eeyye  2 lakki |  | Yoo lakki jette 417tti darbi |
| 416 | Bu'aan isaahoo? | 1 Positive  2 Negative |  |  |
| 417 | Baatii sadan darban keessatti qaamni si dhiigee beekaa? | 1 eeyye  2 lakki |  | Yoo lakki jette 420tti darbi |
| 418 | Yoo eeyye jette dawaa kanaaf fudhatte ni jirtaa? | 1 eeyye  2 lakki |  |  |
| 419 | Dawaa gosa maalii? | Adda baasi ______________ |  |  |
| 420 | Dhukkuba rakkoo kaleen walqabatan kan baatii sadan darban keessatti mana yaalaatti sitti himame ni jiraa? | 1 eeyye  2 lakki |  |  |
| 421 | Sukkaara dhiigakee kan yoo xiqqaate saatii 8f osoon nyaatiin yeroo dhiyootti siif hojjetame ni beektaa (FBS)? | Yooeeyyejette, mg/dl  Yoo lakki jette,kaardii ilaali |  |  |
| 422 | HbA1C (glycosylated hemoglobin) yeroo dhiyootti siif hojjetame ni beekta? | Yoo eeyye jette ___%  Yoo lakki jette ,kaardii ilaali |  |  |

| **Kutaa V: Safartuu Antiropometrii fi bu'aa dhiiga fudhatamee** | | | | |
| --- | --- | --- | --- | --- |
| G501 | Hanga ulfaatinaa | (Kg dhaan) |  |  |
| G502 | Dheerina | (Meetraan) |  |  |
| G503 | Waist circumference | (Cm dhaan) |  |  |
| G504 | Heemoogloobinii argame | (g/dl) |  |  |
| G505 | Dhiibbaa dhiigaa | mm/Hg |  |  |
