# Supplementary material for: Anemia and associated factors among type-2 diabetes mellitus patients attending public hospitals in Harari Region, Eastern Ethiopia
Source: PLoS One. 2019 Dec 5;14(12):e0225725. doi: 10.1371/journal.pone.0225725 (PMC6894806; doi:10.1371/journal.pone.0225725)
Supplement: Attachment — Submitted filename: Response to Reviewers.docx [file pone.0225725.s006.docx]

**Date: 31/Oct/2019**

**To: Editorial Board, PLoS ONE**

**Subject: Response letter on the revised manuscript**

Dear Sir/ Madam,

We hope everything is fine. We appreciate and thank to the academic editor and reviewers for investing their time and energy to review and make comments on our manuscript. It is with great pleasure to receive the invaluable and constructive comments for our manuscript.

As per your request for a separate cover letter, the comments with their point-by-point responses are put below here. In addition, the detailed changes made are highlighted in the “revised manuscript with track changes” by activating the “track changes” feature to easily identify the changes/ improvements. Moreover, the manuscript without track change is prepared. We accepted and tried to incorporate all of the comments provided. Therefore, we are kindly requesting you to review our revised manuscript.

Title: Anemia and associated factors among type-2 diabetes mellitus patients attending public hospitals in Harari Region, Eastern Ethiopia

Journal: PLoS ONE

Submission ID: PONE-D-19-24012

Best regards,

Berhe Gebremichael (BSc, MPH), Lecturer and researcher

Haramaya University, Harar, Ethiopia

***General information:*** *For the comments and questions raised by the academic editor and reviewers, we have uploaded point-by-point response letter, manuscript with track changes, and clean manuscript. In addition, we uploaded a separarte language edited manuscript with track changes as supporting information.*

**1. Comments/questions of academic editor and responses**

**Comment-1:** Please ensure that your manuscript meets PLOS ONE's style requirements, including those for file naming.

**Response:** The revised manuscript has already been modified to PLOS ONE's style

**Comment-2:** Our editorial staff has assessed your submission, and we have concerns about the grammar, usage, and overall readability of the manuscript.  We therefore request that you revise the text to fix the grammatical errors and improve the overall readability of the text before we send it for review. We suggest you have a fluent, preferably native, English-language speaker thoroughly copyedit your manuscript for language usage, spelling, and grammar.

**Response:** The revised manuscript has been copyedited by Dr. Tara D’Ann Wilfong. She is a native English language speaker and is from University of Florida. She is global public health specialist with a medical degree background. The language copyedited manuscript with track changes has been uploaded as supporting information

**Comment-3:** Please provide further details concerning the pretesting of the questionnaire involved in this study, i.e. who was this tested on and on how many?

**Response:** It was conducted on 5% of the questionnaires in DilChora General Hospital which is located in Dire Dawa city, eastern Ethiopia. We incorporated it in the revised manuscript on page 9, lines 178-180

**Comment-4:** Please include captions for your Supporting Information files at the end of your manuscript, and update any in-text citations to match accordingly.

**Response:** Corrected accordingly

**2. Comments/questions of reviewer #1 and responses**

**Comment-1:** . Some exclusions have been applied in the sample recruitment. Please provide the reasoning for that.

**Response:** The reason is it was difficult to interview the seriously ill participants and was difficult to take anthropometric data from individuals who can not sit or stand without support. We included this in the revised manuscript on page 5, line 106-107

**Comment-2:** In the sampling procedure section, the sample size of 410 is planned. But at the end, only 374 (line 209) were analyzed. Please clarify the discrepancy.

**Response:** It was due to lost to follow up from their visit/appointment and refusal not to participate. To compensate this non-responders, we added 10% to the final sample size before starting the data collection. We have included this on page 10, line 215-216.

**Comment-3:** Line 260. The wording “the magnitude of anemia… was 130” reads awkward

**Response:** We have modified it and is on page 17, line 266 in the revised manuscript

**3. Comments/questions of reviewer #2**

**Comment-1:** The authors state that that anemia is a major problem in Ethiopia. It would build the rationale for this study if they are able to reference any data supporting this in the introduction.

**Response:** We have put citations for this information on page 4, line 79

**Comment-2:** The methods require clarification in writing. The authors state that the study population was comprised of all T2DM patients, but they also applied a sampling scheme to select the analysis population. Overall, the data appears to be analyzed appropriately to reach the reported conclusions. For adjusted odds ratios, the authors should be sure to report what these estimates have been adjusted for (i.e. what was included in the multivariate model)

**Response:** We have modified the method section in the revised manuscript, particularly the study population and data analysis sections

**Comment-3:** What data is available regarding the admission reason for the study participants? Are these results generalizable to individuals who have T2DM but were not hospitalized?

**Response:** The hospitals have diabetic clinics where diabetic patients are followed as outpatients. So, we directily collected the data from the patients in the diabetic clinics while the patients come for their follow-up. However, the results of this study could not be generalized for diabetics at community level because the study was conducted at hospital level..

**Comment-4:** In the discussion, the authors report both that physical activity is linked to anemia (line 307), which does not match their results table, and that physical inactivity is linked to anemia (line 337). Please clarify.

**Response:** Thank you for your observation; that is a editorial error. We have now made it consistent in the revised manuscript

**Comment-5:** Editing is needed, particularly with respect to lines: 45 (“having faulty in the blood cells”), 73 (clarify in which cases similar signs and symptoms are observed) and in a number of places in the discussion.

**Response:** We have modified lines 45 and 73. In the revised manuscript, the modifications are on lines (44-45) and (75-77), respectively. For the language errors, we have made the manuscript be reviewed and edited by native English speaker. We have also corrected other technical errors.

=====================**END====================**
